# Intersecting Blood Cytokines With Cholesterol Parameters to Profile Patients With Advanced Solid Tumors Receiving Immune Checkpoint Inhibitors

**DOI:** 10.1097/CJI.0000000000000534

**Published:** 2024-07-11

**Authors:** Giulia Mazzaschi, Fabiana Perrone, Giuseppe Maglietta, Elda Favari, Michela Verzè, Monica Pluchino, Roberta Minari, Federica Pecci, Letizia Gnetti, Nicoletta Campanini, Enrico Maria Silini, Massimo De Filippo, Michele Maffezzoli, Giulia Claire Giudice, Irene Testi, Marcello Tiseo, Federico Quaini, Sebastiano Buti

**Affiliations:** * Medical Oncology Unit, University Hospital of Parma, Parma, Italy; † Department of Medicine and Surgery, University of Parma, Parma, Italy; ‡ Clinical and Epidemiological Research Unit, University Hospital of Parma, Parma, Italy; § Food and Drug Department, University of Parma, Parma, Italy; ‖ Pathology Unit, University Hospital of Parma, Parma, Italy; ¶ Radiology Unit, University Hospital of Parma, Parma, Italy

**Keywords:** cholesterol homeostasis, inflammatory cytokines, immune checkpoint inhibitors, biomarkers, cancer

## Abstract

The study investigated the relationship between serum proinflammatory cytokine levels, cholesterol metabolism, and clinical outcome in cancer patients undergoing immune checkpoint inhibitors (ICIs). Peripheral blood was collected before therapy from ICI-treated advanced cancer patients. We retrospectively assessed plasma total cholesterol (TC), ABCA1- and ABCG1-mediated cholesterol efflux (CE), passive diffusion (PD), cholesterol loading capacity (CLC), and serum IL-6, IL-10, and TNF-α. The association between blood cholesterol parameters and inflammatory cytokines and their effect on overall survival (OS), progression-free survival (PFS), and clinical benefit (CB) from ICIs were statistically assessed. Among 70 consecutively enrolled patients (nonsmall cell lung cancer: 94%; renal cell carcinoma: 6%), TC, CLC, and cholesterol PD resulted significantly higher in IL-6^low^ and IL-10^low^ cases (*P*<0.05), whereas ABCA1-mediated CE was increased in IL-10^high^ patients (*P*=0.018). Uni- and multivariable analysis revealed meaningfully longer OS and PFS in IL-6^low^ (HR 2.13 and 2.97, respectively) and IL-10^low^ (HR 3.17 and 2.62) groups. At univariate analysis all cholesterol-related indices significantly correlated with OS and PFS, whereas at multivariate only high PD was validated as a protection factor (OS, HR 0.75; PFS, HR 0.84). Finally, uni- and multivariable showed a statistically significant inverse association of CB with ABCG1-CE (OR 0.62), as with IL-6 (OR 0.13) and IL-10 (OR 0.10). In-depth characterization of the interplay between blood cholesterol metabolism and immune-inflammatory cytokines might provide novel insights into the complex relationship among cancer, inflammation, lipids profile, and response to immunotherapy.

Immune checkpoint inhibitors (ICIs) have revolutionized the treatment landscape of several solid malignancies, including metastatic renal cell carcinoma (mRCC) and nonsmall cell lung cancer (NSCLC), providing alternatives to targeted therapies or chemotherapy.^1,2^ However, only a limited fraction of patients experiences prolonged clinical benefit from ICIs, highlighting the need to identify prognostic and predictive biomarkers.^3–5^

Several tissue and circulating factors have been proposed as prognostic or predictive determinants of ICIs benefit.^3–6^ Among these, plasma and tissue cholesterol metabolism and cytokines represent promising and valuable variables.

Selected clinical trials have shown a correlation between plasma cholesterol levels and clinical outcome of patients treated with ICIs.^7,8^ Furthermore, intracellular cholesterol modulates tumor-infiltrating lymphocytes (TILs) activity and, through lipid rafts clustering, recruits bioactive molecules and adaptor proteins involved in many oncogenic and immune pathways.^9^

The implication of cholesterol efflux mechanisms on the biology and therapy of mRCC and NSCLC is an area of intense investigation.^10^ As previously described, cells expel cholesterol through 4 main routes: passive diffusion (PD), scavenger receptor class B type I (SR-BI) facilitated diffusion, active efflux by ATP-binding cassette transporter A1 (ABCA1)- and ABCG1-mediated efflux to apoA-1 and high- density lipoprotein (HDL).^11^ There is a close interaction between ABCA1 and ABCG1 transporters and HDL cholesterol activity, measured as cholesterol efflux capacity (CEC), linking cholesterol metabolism with cancer-immune interplay.^12,13^

We investigated indices of cholesterol metabolism (total cholesterol, ABCA1, ABCG1, PD, and cholesterol loading capacity [CLC]) together with clinical outcome and systemic inflammatory status in patients with advanced renal and lung cancer treated with immunotherapy. We have previously provided evidence on the effect of cholesterol serum quality on outcomes of patients with ICI-treated advanced cancer.^14^ Herein, we mainly explored the potential relationship between circulating proinflammatory cytokines levels and cholesterol and their prognostic role in the same setting.

## MATERIAL AND METHODS

### Study Design

We conducted an observational, retrospective, monocentric study aimed at investigating the correlation between indices of cholesterol metabolism (blood total cholesterol, cholesterol efflux: active-mediated CEC, cholesterol PD and CLC) and serum proinflammatory cytokines in a cohort of advanced cancer patients treated with ICIs. The association of CEC, cholesterol PD and CLC with overall survival (OS), progression-free survival (PFS), and clinical benefit represented the object of a previous article.^14^ Here, we tested whether the systemic inflammatory status, as assessed by plasma levels of IL-6, IL-10, and TNF-α, may correlate with cholesterol metabolism and survival outcome.

OS was defined as the time from immunotherapy initiation until death from any cause. PFS was defined as the time from immunotherapy initiation to the first documented tumor progression or death, whichever occurred first. Patients without event occurrence at the data cutoff of December 31, 2021, were considered as censored at the time of the last follow-up. Clinical benefit was defined as the proportion of patients experiencing an objective response (either complete or partial response) or stable disease as best response to immunotherapy according to Response Evaluation Criteria in Solid Tumors version 1.1 (RECIST 1.1).

### Patient Eligibility

This study included advanced cancer patients consecutively treated from October 2013 to October 2018 with single anti-PD-1, anti-PD-L1, or anti-CTLA-4 or with anti-PD-1 plus anti-CTLA-4 combination, regardless of the treatment line, at the Medical Oncology Unit of the University Hospital of Parma (Italy).

Eligible patients satisfied the following criteria: histologically or cytologically confirmed diagnosis of locally advanced or metastatic NSCLC or RCC; at least 1 ICI administration; stored serum sample collected before starting immunotherapy; plasmatic cholesterol level assessed within 1 month before immunotherapy. Patients with incidental acute or chronic inflammatory states or infectious diseases or recent vaccination were excluded.

All patients provided written informed consent to receive ICI treatment. All patients who were alive at the time of the data collection provided informed consent to be included in the study. The procedures followed were in accordance with the Declaration of Helsinki. The study was approved by the local ethical committee (Comitato Etico Area Vasta Emilia Nord; protocol number 37649, approved September 21, 2021).

### Assessment of Cholesterol Homeostasis

CEC, CLC, and PD were measured following a previously described methodology^14^ based on the incubation of patient’s plasma with well-established cell lines (J774 A.1 macrophages, ATCC; Chinese hamster ovary cells) and using standardized isotope techniques ((1,2-3H)-cholesterol, PerkinElmer, Milan, Italy). Reference standard and interassay variability were normalized employing a pool of normolipidemic human sera.^15^ All experiments were performed by experienced personnel at the Department of Food and Drug, University of Parma.

### Measurement of Serum Cytokines

IL-6, IL-10, and TNF-α concentrations were evaluated on stored serum samples with enzyme-linked immunosorbent assay (ELISA) Quantikine Immunoassay (R&D Systems). Aliquots of 50 to 200 μL of serum were used for each assay following the manufacturer’s instructions. On each sample, the ELISA test for cytokines determination was carried in duplicate to increase the reproducibility of the results. These analyses were performed at the laboratory of the Oncology Unit, University Hospital of Parma. Cutoff values of cytokines used for correlations with cholesterol parameters and clinical outcome were based on data from literature^16,17^ and established as follows: IL-6=11 pg/mL, IL-10=6 pg/mL, and TNF-α=22 pg/mL, respectively.

### Statistical Analysis

Due to the observational retrospective nature of the study and given that the primary and secondary endpoints were to explore the correlations between IL-6, IL-10, TNF-α, and cholesterol parameters with clinical outcomes, the sample size was not determined by a formal statistical estimation. Baseline characteristics were summarized by using descriptive statistical metrics, as median and interquartile range (IQR), and absolute and relative frequencies for quantitative and categorical variables, respectively. Boxplots were adopted to graphically represent the distribution of CEC, ABCA1, and ABCG1 cholesterol efflux, cholesterol PD and CLC according to high versus low IL-6, IL-10, and TNF-α levels based on a priori threshold from the literature.^16,17^ Wilcoxon test was used to perform comparison among groups. Kaplan–Meier curves were employed to graphically illustrate the association of the IL-6, IL-10, and TNF-α with 1-year patient survival outcomes (OS and PFS). Univariable and multivariable semiparametric Cox regression models were implemented to measure associations of cholesterol parameters and cytokines with survival outcomes; the logistic regression model was used to assess the associations with clinical benefit. Multivariable models included all the cholesterol- and cytokine-related parameters regardless their statistical significance on univariable analysis. All statistical analyses were performed using R Statistical Software v. 4.2.2.

## RESULTS

Overall, 70 patients were enrolled in the study, of which 2 were excluded due to inadequate serum sampling for biochemical determinations. The median age was 72 years (range: 41–84 y) with male predominance (67.1%). The primary tumors mostly consisted of NSCLC (94.2%), whereas kidney cancer accounted for only 5.8%. Twenty-seven patients (38.5%) had 2 or less metastatic sites at the time of diagnosis, whereas 43 (61.5%) displayed more than 2 metastatic lesions. Most patients received immunotherapy as second or subsequent lines (84.3%), and 11 (15.7%) underwent immunotherapy as first-line treatment (Table 1).

**TABLE 1:** Clinicopathological Characteristics of the Overall Population

Clinicopathological Variables	Patients, n=70
Age, median (range)	72 (41–84)
	n (%)
Sex	
Male	47 (67.1)
Female	23 (32.9)
Smoking status	
Current	21 (30.0)
Former	28 (40.0)
Never	21 (30.0)
ECOG PS	
0	23 (32.9)
1	38 (54.3)
2	9 (12.8)
Primary tumors	
Kidney	4 (5.8)
NSCLC	66 (94.2)
ADC	43 (65.1)
Non-ADC	19 (28.8)
NSCLC NOS	4 (6.1)
No. of Metastatic sites involved	
1	5 (7.1)
2	22 (31.4)
3	20 (28.6)
4	8 (11.4)
≥5	15 (21.4)
No. of previous lines of therapy	
<1	11 (15.7)
≥1	59 (84.3)
ICI administered	
anti-PD-1	62 (88.6)
anti PD-L1	6 (8.6)
anti PD-1/PD-L1 + anti-CTLA-4	2 (2.8)
Drug administered	
Nivolumab	48 (68.6)
Pembrolizumab	14 (20.0)
Atezolizumab	6 (8.6)
Nivolumab + ipilimumab	2 (2.8)
History of hypercholesterolemia	
Yes	24 (34.3)
No	46 (65.7)
Total cholesterol levels, mg/dL	
<200	54 (77.1)
≥200	16 (22.9)
History of hypertriglyceridemia	
Yes	11 (15.7)
No	58 (82.8)
NA	1 (1.5)
Statin therapy	
Yes	21 (30.0)
No	49 (70.0)
BMI, kg/m^2^	
<25	43 (61.4)
≥25	27 (38.6)

ADC indicates adenocarcinoma; BMI, body mass index; CTLA 4, cytotoxic T lymphocyte antigen 4; ECOG PS, Eastern Cooperative Oncology Group Performance Status; ICI, immune checkpoint inhibitor; NOS, not otherwise specified; NSCLC, nonsmall cell lung cancer; PD-1, programmed death-1; PD-L1, programmed death ligand-1.

At the date of December 31, 2021, 60 patients died and 61 experienced disease progression.

### Association Between Cytokines and Cholesterol Parameters

**FIGURE 1:**
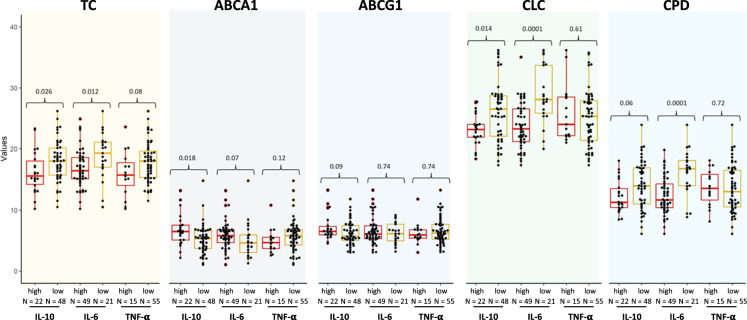
Box plot documenting the distribution of TC, ABCA1- and ABCG1-mediated cholesterol efflux, CLC and cholesterol PD according to high versus low cytokine levels (see text for the thresholds). ABCA1, ATP-binding cassette transporter A1; ABCG1, ATP-binding cassette transporter G1; CLC, cholesterol loading capacity; CPD, cholesterol passive diffusion; IL-6, interleukin 6; IL-10, interleukin 10; TC, total cholesterol; TNF-α, tumor necrosis factor-α.

Indices of cholesterol metabolism were significantly associated to cytokine levels, especially IL-6 and IL-10, whereas TNF-α was less effectful (Fig. 1). Specifically, total cholesterol plasmatic levels were significantly higher in IL-6^low^ (*P*=0.012) and IL-10^low^ (*P*=0.026) patients, whereas only a trend was seen in TNF-α^low^ cases (*P*=0.08). Cholesterol efflux mediated by ABCA1 was also significantly increased in IL-10^high^ (*P*=0.018) and to a lesser extent in IL-6^high^ (*P*=0.07) patients. A direct positive correlation between ABCG1-mediated cholesterol efflux and IL-10 was observed, although not reaching statistical significance (*P*=0.09), whereas CLC was inversely correlated with IL-10 (*P*=0.014) and IL-6 (*P*=0.001) levels. Likewise, cholesterol PD was significantly higher in IL-6^low^ (*P*=0.0001) and a similar trend was present in IL-10^low^ (*P*=0.06) cases.

### Effect of Cytokines and Cholesterol Parameters on Clinical Outcomes

**FIGURE 2:**
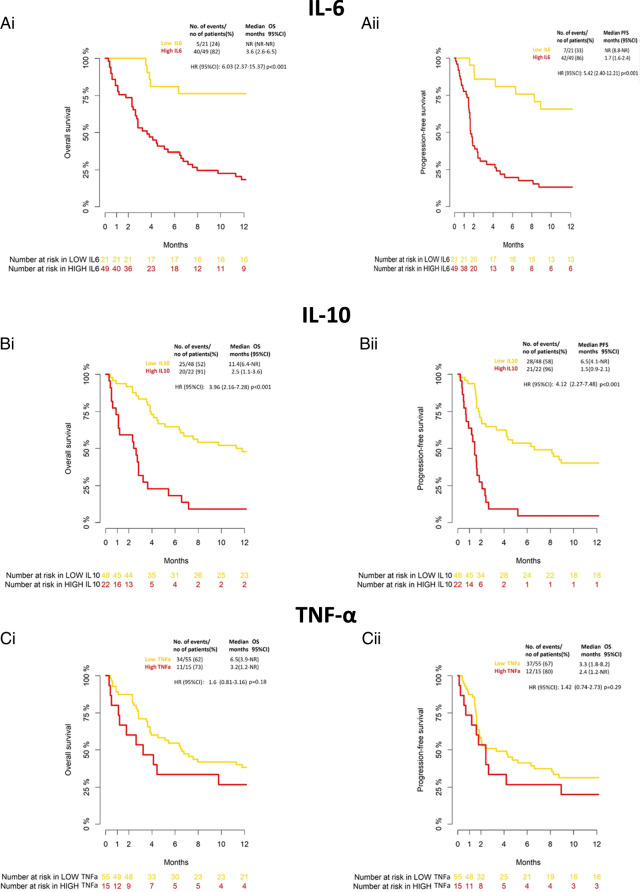
Overall survival (OS) and progression-free survival (PFS) in ICIs treated patients with advanced cancer according to IL-6 (Ai,ii), IL-10 (Bi,ii), and TNF-α (Ci,ii) levels. Numbers at risk are reported at the bottom of Kaplan–Meier curves. CI, confidence interval; HR, hazard ratio; IL-6, interleukin 6; IL-10, interleukin 10; NR, not reached; TNF-α, tumor necrosis factor-α.

As reported in Figure 2, serum cytokine concentrations were significantly associated with clinical outcome in terms of both OS and PFS. Specifically, low IL-6 and IL-10 levels appeared to condition a significantly prolonged OS [IL-6, low vs. high: median OS (mOS)=NR vs. 3.6 mo, *P*<0.001; IL-10, low vs. high: mOS=11.4 vs 2.5 mo, *P*<0.001], and a parallel trend was seen for TNF-α, although without reaching statistical significance (*P*=0.18) (Figs. 2Ai, Bi, Ci).

Similarly, patients with low baseline IL-6 and IL-10 displayed longer PFS [IL-6, low vs. high: median PFS (mPFS)=NR vs. 1.7 mo, *P*<0.001; IL-10, low vs. high: mPFS=6.5 vs. 1.5 mo, *P*<0.001], whereas TNF-α did not show a striking effect (*P*=0.29) (Figs. 2Aii, Bii, Cii).

At univariable analysis, all cholesterol-related features and IL-10 and IL-6 levels were significantly associated with OS (Table 2). Multivariable analysis confirmed an independent direct correlation between cholesterol PD and OS [HR 0.75 (95% CI 0.63–0.9), *P*=0.001] and an inverse correlation between IL-10 and OS [HR 3.17 (95% CI 1.48–6.79), *P*=0.003]. On multivariable model, an inverse correlation between TNF-α and OS also emerged [HR 2.38 (95% CI 1.05–5.43), *P*=0.038], whereas that between IL-6 and OS was not preserved.

**TABLE 2:** Cox Regression Models for OS and PFS

	Univariable	Multivariable
	HR [95% CI], *P*	HR [95% CI], *P*
**OS**		
TC*	0.99 [0.98–0.99], **0.02**	1.01 [0.99–1.02], 0.535
ABCA1 cholesterol efflux*	1.19 [1.09–1.29], **<0.001**	1.05 [0.9–1.23], 0.534
ABCG1 cholesterol efflux*	1.21 [1.06–1.38], **0.004**	1.04 [0.91–1.19], 0.534
Cholesterol PD*	0.72 [0.64–0.80], **<0.001**	0.75 [0.63–0.9], **0.001**
CLC*	0.83 [0.77–0.89], **<0.001**	0.95 [0.85–1.07], 0.393
IL-6 (high vs low)	6.03 [2.37–15.37], **<0.001**	2.13 [0.71–6.38], 0.175
IL-10 (high vs low)	3.96 [2.16–7.28], **<0.001**	3.17 [1.48–6.79], **0.003**
TNF-α (high vs low)	1.60 [0.81–3.16], 0.18	2.38 [1.05–5.43], **0.038**
**PFS**		
TC*	0.99 [0.98–0.99], **0.01**	0.99 [0.99–1.01], 0.679
ABCA1 cholesterol efflux*	1.12 [1.02–1.23], **0.01**	0.99 [0.841–.16], 0.878
ABCG1 cholesterol efflux*	1.19 [1.04–1.36], **0.01**	1.06 [0.92–1.23], 0.398
Cholesterol PD*	0.80 [0.73–0.88], **<0.001**	0.84 [0.72–0.97], **0.021**
CLC*	0.88 [0.82–0.94], **<0.001**	1.02 [0.92–1.14], 0.695
IL-6 (high vs low)	5.42 [2.40–12.21], **<0.001**	2.97 [1.14–7.73], **0.025**
IL-10 (high vs low)	4.12 [2.27–7.48], **<0.001**	2.62 [1.31–5.23], **0.006**
TNF-α (high vs low)	1.42 [0.74–2.73], 0.29	1.18 [0.56–2.51], 0.660

* All the variables were considered as continuous.

Bold values are statically significance *P* < 0.05.

ABCA1 indicates ATP-binding cassette transporter A1; ABCG1, ATP-binding cassette transporter G1; CI, confidence interval; CLC, cholesterol loading capacity; HR, hazard ratio; IL-10, interleukin 10; IL-6, interleukin 6; OS, overall survival; PD, passive diffusion; PFS, progression-free survival; TC, total cholesterol; TNF-α, tumor necrosis factor-α.

The univariable analysis for PFS validated the effect of all cholesterol parameters as well as of IL-10 and IL-6 (Table 2). When challenged on multivariable models, cholesterol PD was confirmed as a protection factor [HR 0.84 (95% CI 0.72–0.97), *P*=0.021], and a relevant inverse correlation between PFS and IL-6 [HR 2.97 (95% CI 1.14–7.73), *P*=0.025] and IL-10 [HR 2.62 (95% CI 1.31–5.23), *P*=0.006] was corroborated (Table 2).

Finally, we tested the prognostic effect of blood immune-inflammatory and metabolic descriptors on positive clinical outcome from ICI treatment. At univariable analysis, except for ABCA1-mediated efflux, all measured cholesterol parameters and IL-6 and IL-10 significantly affected clinical benefit from ICIs (Table 3). Importantly, the negative effect on ICI efficacy exerted by ABCG1 cholesterol efflux [OR 0.62 (95% CI 0.36–0.95), *P*=0.048], IL-6 [OR 0.13 (95% CI 0.02–0.73), *P*=0.026], and IL-10 [OR 0.10 (95% CI 0.01–0.58), *P*=0.018] was confirmed at multivariable model.

**TABLE 3:** Cox Regression Models for CB

	Univariable	Multivariable
CB	OR [95% CI], *P*	OR [95% CI], *P*
TC*	1.02 [1.00–1.03], **0.045**	1.00 [0.97–1.02], 0.808
ABCA1 cholesterol efflux*	0.82 [0.65–1.00], 0.068	1.16 [0.82–1.64], 0.395
ABCG1 cholesterol efflux*	0.67 [0.48–0.88], **0.009**	0.62 [0.36–0.95], **0.048**
Cholesterol PD*	1.31 [1.13–1.57], **0.001**	1.10 [0.84–1.46], 0.486
CLC*	1.30 [1.14–1.54], **<0.001**	1.08 [0.85–1.4], 0.514
IL-6 (high vs low)	0.07 [0.01–0.23], **<0.001**	0.13 [0.02–0.73], **0.026**
IL-10 (high vs low)	0.06 [0.01–0.24], **<0.001**	0.10 [0.01–0.58], **0.018**
TNF-α (high vs low)	0.97 [0.93–1.01], 0.22	0.75 [0.11–4.8], 0.762

* All the variables were considered as continuous.

Bold values are statically significance *P* < 0.05.

ABCA1 indicates ATP-binding cassette transporter A1; ABCG1, ATP-binding cassette transporter G1; CB, clinical benefit; CI, confidence interval. CLC, cholesterol loading capacity; HR, hazard ratio; IL-10, interleukin 10; IL-6, interleukin 6; PD, passive diffusion; TC, total cholesterol; TNF-α, tumor necrosis factor-α.

## DISCUSSION

In this study, we explored the correlation between serum cytokines levels and cholesterol metabolism on oncological outcomes in patients with advanced or metastatic NSCLC and RCC treated with ICIs.

The hypothesis that cytokines could be valuable prognostic and predictive biomarkers has been already investigated and validated by other studies.^18–20^ Accordingly, the present data corroborate a negative effect of high levels of proinflammatory cytokines on survival and benefit from ICIs.

We previously showed a positive correlation between cholesterol PD and clinical outcomes.^14^ This association was confirmed and strengthened here after adjustment for the confounding effect of cytokines in the multivariable analyses. In addition, we demonstrated that patients with high cytokine levels displayed lower TC and an enhanced ABCA1 and ABCG1 cholesterol efflux, at the expense of cholesterol PD.

It is known that an acute inflammatory state sustained by several cytokines, including IL-6, can induce a reduction in LDL cholesterol levels.^21^ Furthermore, the results from the *InChianti* study, a prospective population-based study of aged individuals, highlighted an inverse correlation between plasma levels of IL-6 and HDL cholesterol, although a cause–effect relationship was not identified.^22^ Similarly, the present data cannot provide a mechanistic link between increased cytokines and lower TC, cholesterol PD, and CLC, nevertheless an association was supported here. A potential mechanism of this inverse relationship has been provided in vitro on human endothelial cells by the documentation of the inhibitory effect of human plasma HDL cholesterol on both spontaneous or TNF-α-induced IL-6 production at RNA and protein levels.^23^

Patients with the best clinical outcomes following ICIs were those with low cytokine levels, low activation of ABCA1- and ABCG1-mediated cholesterol efflux, and high cholesterol PD. There are different types of HDL particles involved in cholesterol efflux mechanisms. Specifically, ABCA1 and ABCG1 transporters interact with HDL particles that are small and poor in cholesterol.^24^ Considering the inverse relationship between active and passive cholesterol transport, passive cholesterol efflux could reflect the presence of larger and cholesterol-rich HDL particles. We speculate that larger HDL particles might be able to remove excess cholesterol, stored in the tumor microenvironment, which is responsible for the exhaustion of CD8 cells.^25,26^ Moreover, as recently documented, HDL appeared to possess both antioxidative^27^ and antiangiogenic^28^ properties and to significantly interact with T CD4^+^ cells^29^; this might explain their antitumor activity. High levels of cholesterol PD and its association with low IL-6 and IL-10 levels, could reflect a low systemic inflammatory state (or a “good” chronic inflammation state) and might favorably condition the proficiency of the tumor microenvironment to respond to ICIs. These contentions are largely in line with the critical implication of cholesterol homeostasis on functional aspects of the immune response to cancer.^30,31^

In agreement with the literature, we documented high levels of IL-6, endowed with immunostimulatory activity, and IL-10, with immunoinhibitory properties, as strong negative prognostic factors. Conversely, due to its pleiotropic function (as cytokine and adipokine) and conflicting role in cancer,^32^ TNF-α concentrations had no prognostic effect. The limited therapeutic efficacy of TNF-α inhibition in cancer settings might support, at least in part, this finding.^33,34^

Our observations have potential implications on new therapeutic scenarios. Antibodies directed against cytokines, in addition to counteracting the systemic inflammatory state, might restore cholesterol metabolic homeostasis, ultimately preventing the functional exhaustion of immune cells and promoting their reinvigoration by ICIs. In favor of this hypothesis, the work by Moraitis and colleagues on patients with B-cell-related disorders, including lymphomas, highlighted a strong inverse correlation between IL-10 and HDL cholesterol.^35^ By virtue of this inverse correlation, they speculated the existence of a cause–effect relationship based on the results of a randomized controlled trial involving patients with psoriatic arthritis and receiving recombinant human IL-10.^35,36^ Finally, as reviewed by Yu et al,^37^ several clinical trials are ongoing in the attempt to foster anti-cancer immunity by acting on lipid metabolism. Importantly, interference with cholesterol efflux by Liver X Receptor (LXR) agonists or with TNF-α, IL-6, and IL-10 by fatty acid synthase (FASN) inhibitors are among the lipid-oriented strategies currently translated into clinical setting.^38,39^

Limitations of this study are the retrospective design, the relatively small sample size, the potential selection bias related to the availability of a stored serum as inclusion criteria and the heterogeneity of our patient population in terms of lines of immunotherapy and primary tumor. Nonetheless, our patient cohort is representative of a real-life population and the validated effect of specific cholesterol parameters on clinical outcome indicates the robustness of our previous investigation.

Further studies are warranted to thoroughly explore the cholesterol-cytokines-immunity axis and its role on cancer management in the era of immunotherapy.
